# 2-(4-Fluoro-2-nitro­phen­yl)-4-hy­droxy-9-phenyl­sulfonyl-9*H*-carbazole-3-carbaldehyde

**DOI:** 10.1107/S1600536814002633

**Published:** 2014-02-12

**Authors:** S. Gopinath, K. Sethusankar, Velu Saravanan, Arasambattu K. Mohanakrishnan

**Affiliations:** a Department of Physics, RKM Vivekananda College (Autonomous), Chennai 600 004, India; b Department of Organic Chemistry, University of Madras, Maraimalai Campus, Chennai 600 025, India

## Abstract

In the title compound, C_25_H_15_FN_2_O_6_S, the carbazole ring system is essentially planar, with a maximum deviation of 0.1534 (16) Å for the C atom connected to the 4-fluoro-2-nitro­phenyl ring. It is almost orthogonal to the phenyl­sulfonyl and nitro­phenyl rings, making dihedral angles of 88.45 (8) and 79.26 (7)°, respectively. The mol­ecular structure is stabilized by O—H⋯O and C—H⋯O hydrogen bonds, which generate three *S*(6) ring motifs. In the crystal, mol­ecules are linked by two C—H⋯O hydrogen bonds, which generate *C*(6) and *C*(9) chains running in the [100] and [010] directions, respectively, so forming a two-dimensional network lying parallel to (001). There are also supra­molecular *R*_4_^3^(28) graph-set ring motifs enclosed within these networks.

## Related literature   

For the biological activity and uses of carbazole derivatives, see: Itoigawa *et al.* (2000 ▶); Ramsewak *et al.* (1999 ▶). For their electronic properties and applications, see: Friend *et al.* (1999 ▶); Zhang *et al.* (2004 ▶). For a related structure, see: Gopinath *et al.* (2013 ▶). For bond-length data, see: Allen *et al.* (1987 ▶). For graph-set notation, see: Bernstein *et al.* (1995 ▶). For the the Thrope–Ingold effect, see: Bassindale (1984 ▶).
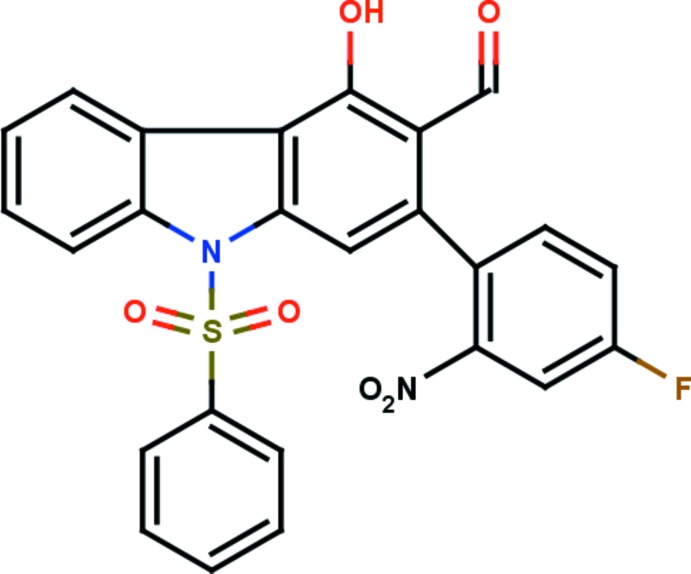


## Experimental   

### 

#### Crystal data  

C_25_H_15_FN_2_O_6_S
*M* *_r_* = 490.45
Monoclinic, 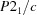

*a* = 8.124 (5) Å
*b* = 14.191 (5) Å
*c* = 18.607 (5) Å
β = 93.820 (5)°
*V* = 2140.4 (16) Å^3^
*Z* = 4
Mo *K*α radiation
μ = 0.21 mm^−1^
*T* = 296 K
0.35 × 0.30 × 0.25 mm

#### Data collection  

Brukker Kappa APEXII CCD diffractometer
Absorption correction: multi-scan (*SADABS*; Bruker, 2008 ▶) *T*_min_ = 0.930, *T*_max_ = 0.949
31082 measured reflections
7207 independent reflections
4725 reflections with *I* > 2σ(*I*)
*R*_int_ = 0.035

#### Refinement  

*R*[*F* ^2^ > 2σ(*F* ^2^)] = 0.048
*wR*(*F* ^2^) = 0.143
*S* = 1.01
7207 reflections
316 parameters
H-atom parameters constrained
Δρ_max_ = 0.32 e Å^−3^
Δρ_min_ = −0.44 e Å^−3^

### 

Data collection: *APEX2* (Bruker, 2008 ▶); cell refinement: *SAINT* (Bruker, 2008 ▶); data reduction: *SAINT*; program(s) used to solve structure: *SHELXS97* (Sheldrick, 2008 ▶); program(s) used to refine structure: *SHELXL97* (Sheldrick, 2008 ▶); molecular graphics: *ORTEP-3 for Windows* (Farrugia, 2012 ▶) and *Mercury* (Macrae *et al.*, 2008 ▶); software used to prepare material for publication: *SHELXL97* and *PLATON* (Spek, 2009 ▶).

## Supplementary Material

Crystal structure: contains datablock(s) global, I. DOI: 10.1107/S1600536814002633/su2695sup1.cif

Structure factors: contains datablock(s) I. DOI: 10.1107/S1600536814002633/su2695Isup2.hkl

Supporting information file. DOI: 10.1107/S1600536814002633/su2695Isup3.cml

CCDC reference:

Additional supporting information:  crystallographic information; 3D view; checkCIF report

## Figures and Tables

**Table 1:** Hydrogen-bond geometry (Å, °)

*D*—H⋯*A*	*D*—H	H⋯*A*	*D*⋯*A*	*D*—H⋯*A*
O1—H1⋯O2	0.82	1.89	2.611 (3)	146
C2—H2⋯O3	0.93	2.37	2.956 (3)	121
C9—H9⋯O4	0.93	2.30	2.902 (3)	122
C18—H18⋯O4^i^	0.93	2.55	3.221 (3)	129
C13—H13⋯O4^ii^	0.93	2.50	3.337 (3)	150

Symmetry codes: (i) 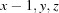; (ii) 
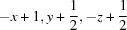
.

**Fig. 1.**
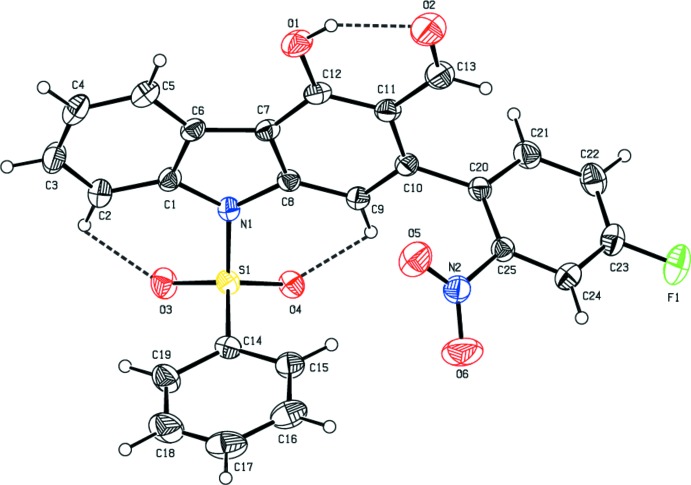
The molecular structure of the title molecule, with atom labelling. Displacement ellipsoids are drawn at 30% probability level. The O—H···O and C—H···O hydrogen bonds are shown as dashed lines [see Table 1 for details].

**Fig. 2.**
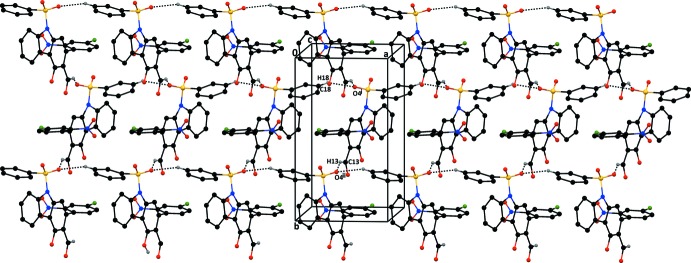
The crystal packing of the title compound viewed along the a axis. The C—H···O hydrogen bonds are shown as dashed lines [see Table 1 for details; symmetry codes: (i) x-1, y, z; (ii) -x+1, y+1/2, -z+1/2].

## supplementary crystallographic information

### 1. Comment

Carbazole and its derivatives have become attractive compounds owing to their applications in pharmacy and molecular electronics. It has been reported that carbazole derivatives exhibit various biological activities such as antitumor and antioxidative (Itoigawa *et al.*, 2000), and anti-inflammatory and antimutagenic (Ramsewak *et al.*, 1999). They also exhibit electroactivity and luminescence and are considered to be potential candidates for electronic applications such as colour displays, organic, semiconductors, laser and solar cells (Friend *et al.*, 1999; Zhang *et al.*, 2004).

The title compound, Fig. 1, comprises a carbazole ring system which is attached to a phenylsulfonyl ring, a nitrophenyl ring, a carbaldehyde group and a hydroxyl group. The carbazole ring system is essentially planar with maximum deviation of 0.1534 (16) Å for the carbon atom C10. The atom O1 significantly deviates from the carbazole ring by 0.1845 (15) Å. The carbazole ring system is almost orthogonal to the phenyl ring attached to the sulfonyl group and the nitrophenyl ring with dihedral angles of 88.45 (8)° and 79.26 (7)°, respectively.

As a result of electron-withdrawing character of the phenylsulfonyl group, the bond lengths N1–C1 = 1.427 (2) Å and N1–C8 = 1.412 (2) Å are longer than the mean value of 1.355 (14) Å (Allen *et al.*, 1987). Atom S1 has a distorted tetrahedral configuration. The widening of angle O3—S1—O4 [119.96 (7)°] and narrowing of angle N1—S1—C14 [104.20 (7) °] from the ideal tetrahedral value are attributed to the Thrope-Ingold effect (Bassindale *et al.*, 1984).

The sum of the bond angles around atom N1 [356.77°] indicate sp^2^ hybridization. The benzene ring (C20–C25) is almost coplanar with the nitro group and the fluorine atom with torsion angles C21–C20–C25–N2 [-179.64 (16) °] and C21–C22–C23–F1 [179.40 (18) °].

The molecular structure is stabilized by O—H···O and C—H···O hydrogen bonds (Table 1 and Fig. 1), which generate three S(6) ring motifs (Bernstein *et al.*, 1995).

In the crystal, molecules are linked by C-H···O hydrogen bonds, which generate *C*(6) and *C*(9) chains running in the directions [1 0 0] and [0 1 0] respectively (Table 1 and Fig 2), and form a two dimensional network lying parallel to (0 0 1). There are also *R*^3^_4_(28) supramolecular graph-set ring motifs enclosed within these networks. The symmetry codes are: (i) -1 + *x*, *y*, *z* (ii) 1 - *x*, 1/2 + *y*, 1/2 - *z*.

### 2. Experimental

To a solution of 2-(4-fluoro-2-nitrophenyl)-4-methoxy-9-(phenylsulfonyl)-9H-carbazole-3-cabaldehyde (0.76 g, 1.5 mmol) in dry DCM (20 mL), 1*M* solution of BBr3 (1.65 mL, 1.65 mmol) in DCM was added at 273 K. After completion of the reaction (monitored by TLC), the mixture was poured into ice water (50 mL) containing HCl (5 mL). The organic layer was seperated and the aqueous layer was then extracted with DCM (2 × 10 ml). The combined organic layers were washed with water (2 × 30 ml) and dried (NaSO4). Removal of the solvent followed by tituration of the crude product with MeOH (10 mL) afforded the title compound as a pale yellow solid (0.73 g, 96%; M.p. 501–503 K). Block-like yellow crystals were obtained by slow evaporation of a solution in methanol.

### 3. Refinement

The H atoms were localized from difference electron-density maps. They were refined as riding atoms with their distances geometrically constrained: O-H = 0.82 Å with *U*_iso_(H) = 1.5*U*_eq_(O), and C—H = 0.93 Å with *U*_iso_(H) = 1.2U_eq_(C).

### Crystal data

**Table:**

C_25_H_15_FN_2_O_6_S	*F*(000) = 1008
*M**_r_* = 490.45	*D*_x_ = 1.522 Mg m^−^^3^
Monoclinic, *P*2_1_/*c*	Mo *K*α radiation, λ = 0.71073 Å
Hall symbol: -P 2ybc	Cell parameters from 7207 reflections
*a* = 8.124 (5) Å	θ = 2.2–31.7°
*b* = 14.191 (5) Å	µ = 0.21 mm^−^^1^
*c* = 18.607 (5) Å	*T* = 296 K
β = 93.820 (5)°	Block, yellow
*V* = 2140.4 (16) Å^3^	0.35 × 0.30 × 0.25 mm
*Z* = 4	

### Data collection

**Table:**

Brukker Kappa APEXII CCD diffractometer	7207 independent reflections
Radiation source: fine-focus sealed tube	4725 reflections with *I* > 2σ(*I*)
Graphite monochromator	*R*_int_ = 0.035
ω & φ scans	θ_max_ = 31.7°, θ_min_ = 2.2°
Absorption correction: multi-scan (*SADABS*; Bruker, 2008)	*h* = −11→11
*T*_min_ = 0.930, *T*_max_ = 0.949	*k* = −20→20
31082 measured reflections	*l* = −26→27

### Refinement

**Table:**

Refinement on *F*^2^	Primary atom site location: structure-invariant direct methods
Least-squares matrix: full	Secondary atom site location: difference Fourier map
*R*[*F*^2^ > 2σ(*F*^2^)] = 0.048	Hydrogen site location: inferred from neighbouring sites
*wR*(*F*^2^) = 0.143	H-atom parameters constrained
*S* = 1.01	*w* = 1/[σ^2^(*F*_o_^2^) + (0.0698*P*)^2^ + 0.4156*P*] where *P* = (*F*_o_^2^ + 2*F*_c_^2^)/3
7207 reflections	(Δ/σ)_max_ = 0.001
316 parameters	Δρ_max_ = 0.32 e Å^−^^3^
0 restraints	Δρ_min_ = −0.44 e Å^−^^3^

### Special details

**Table:**

Geometry. All e.s.d.'s (except the e.s.d. in the dihedral angle between two l.s. planes) are estimated using the full covariance matrix. The cell e.s.d.'s are taken into account individually in the estimation of e.s.d.'s in distances, angles and torsion angles; correlations between e.s.d.'s in cell parameters are only used when they are defined by crystal symmetry. An approximate (isotropic) treatment of cell e.s.d.'s is used for estimating e.s.d.'s involving l.s. planes.
Refinement. Refinement of *F*^2^ against ALL reflections. The weighted *R*-factor *wR* and goodness of fit *S* are based on *F*^2^, conventional *R*-factors *R* are based on *F*, with *F* set to zero for negative *F*^2^. The threshold expression of *F*^2^ > σ(*F*^2^) is used only for calculating *R*-factors(gt) *etc*. and is not relevant to the choice of reflections for refinement. *R*-factors based on *F*^2^ are statistically about twice as large as those based on *F*, and *R*- factors based on ALL data will be even larger.

### Fractional atomic coordinates and isotropic or equivalent isotropic displacement parameters (Å^2^)

**Table:**

	*x*	*y*	*z*	*U*_iso_*/*U*_eq_	
C1	0.22774 (18)	0.88060 (12)	−0.01369 (8)	0.0374 (3)	
C2	0.1387 (2)	0.83895 (14)	−0.07118 (9)	0.0495 (4)	
H2	0.1307	0.7738	−0.0757	0.059*	
C3	0.0621 (2)	0.89880 (16)	−0.12166 (10)	0.0566 (5)	
H3	−0.0006	0.8729	−0.1604	0.068*	
C4	0.0754 (2)	0.99483 (15)	−0.11661 (10)	0.0552 (5)	
H4	0.0208	1.0325	−0.1514	0.066*	
C5	0.1683 (2)	1.03627 (14)	−0.06079 (9)	0.0482 (4)	
H5	0.1798	1.1014	−0.0582	0.058*	
C6	0.24489 (19)	0.97816 (12)	−0.00809 (8)	0.0378 (3)	
C7	0.33800 (19)	0.99774 (11)	0.05859 (8)	0.0361 (3)	
C8	0.37439 (17)	0.91229 (10)	0.09332 (8)	0.0338 (3)	
C9	0.45053 (19)	0.90720 (11)	0.16234 (8)	0.0368 (3)	
H9	0.4734	0.8494	0.1843	0.044*	
C10	0.49084 (19)	0.99004 (11)	0.19702 (8)	0.0366 (3)	
C11	0.4656 (2)	1.07785 (11)	0.16215 (9)	0.0412 (4)	
C12	0.3891 (2)	1.08113 (11)	0.09237 (9)	0.0408 (4)	
C13	0.5105 (3)	1.16400 (13)	0.19907 (11)	0.0577 (5)	
H13	0.5595	1.1591	0.2455	0.069*	
C14	0.10618 (19)	0.73000 (10)	0.11778 (9)	0.0380 (3)	
C15	0.1077 (2)	0.76246 (12)	0.18780 (10)	0.0470 (4)	
H15	0.2061	0.7803	0.2125	0.056*	
C16	−0.0402 (3)	0.76793 (15)	0.22048 (13)	0.0623 (5)	
H16	−0.0425	0.7906	0.2673	0.075*	
C17	−0.1846 (3)	0.73945 (18)	0.18295 (16)	0.0730 (7)	
H17	−0.2839	0.7433	0.2049	0.088*	
C18	−0.1833 (3)	0.70603 (18)	0.11453 (15)	0.0729 (7)	
H18	−0.2812	0.6863	0.0905	0.087*	
C19	−0.0383 (2)	0.70121 (14)	0.08066 (12)	0.0558 (5)	
H19	−0.0374	0.6790	0.0337	0.067*	
C20	0.55668 (19)	0.98415 (11)	0.27357 (8)	0.0367 (3)	
C21	0.7260 (2)	0.98788 (14)	0.29145 (10)	0.0503 (4)	
H21	0.7975	0.9992	0.2554	0.060*	
C22	0.7897 (2)	0.97524 (15)	0.36099 (11)	0.0550 (5)	
H22	0.9028	0.9789	0.3721	0.066*	
C23	0.6842 (2)	0.95715 (13)	0.41362 (10)	0.0500 (4)	
C24	0.5168 (2)	0.95119 (12)	0.39949 (10)	0.0460 (4)	
H24	0.4468	0.9375	0.4356	0.055*	
C25	0.45671 (19)	0.96635 (11)	0.32961 (8)	0.0367 (3)	
N1	0.31385 (16)	0.83887 (9)	0.04768 (7)	0.0369 (3)	
N2	0.27697 (18)	0.96048 (12)	0.31633 (8)	0.0497 (4)	
O1	0.35888 (18)	1.16282 (9)	0.05773 (7)	0.0589 (4)	
H1	0.3946	1.2066	0.0830	0.088*	
O2	0.4894 (3)	1.24286 (10)	0.17434 (9)	0.0816 (5)	
O3	0.26853 (16)	0.67320 (8)	0.01041 (7)	0.0514 (3)	
O4	0.42270 (13)	0.70889 (8)	0.12536 (6)	0.0419 (3)	
O5	0.20964 (17)	1.00982 (13)	0.27085 (9)	0.0735 (5)	
O6	0.2029 (2)	0.90511 (16)	0.35160 (11)	0.1023 (7)	
F1	0.74611 (17)	0.94386 (10)	0.48170 (7)	0.0770 (4)	
S1	0.28971 (5)	0.72821 (3)	0.07403 (2)	0.03511 (11)	

### Atomic displacement parameters (Å^2^)

**Table:**

	*U*^11^	*U*^22^	*U*^33^	*U*^12^	*U*^13^	*U*^23^
C1	0.0366 (7)	0.0465 (8)	0.0296 (7)	−0.0002 (6)	0.0053 (6)	0.0006 (6)
C2	0.0556 (10)	0.0548 (10)	0.0374 (9)	−0.0056 (8)	−0.0022 (7)	−0.0026 (7)
C3	0.0590 (11)	0.0758 (14)	0.0339 (9)	−0.0018 (10)	−0.0047 (8)	0.0001 (9)
C4	0.0575 (11)	0.0727 (13)	0.0352 (9)	0.0114 (9)	0.0013 (8)	0.0106 (8)
C5	0.0543 (10)	0.0519 (10)	0.0392 (9)	0.0066 (8)	0.0093 (8)	0.0095 (7)
C6	0.0378 (8)	0.0447 (8)	0.0319 (8)	0.0003 (6)	0.0092 (6)	0.0038 (6)
C7	0.0382 (8)	0.0376 (7)	0.0333 (8)	−0.0016 (6)	0.0089 (6)	0.0028 (6)
C8	0.0331 (7)	0.0355 (7)	0.0333 (7)	−0.0034 (5)	0.0059 (6)	−0.0021 (6)
C9	0.0406 (8)	0.0347 (7)	0.0349 (8)	−0.0031 (6)	0.0017 (6)	0.0009 (6)
C10	0.0386 (8)	0.0391 (8)	0.0325 (8)	−0.0048 (6)	0.0066 (6)	−0.0027 (6)
C11	0.0521 (9)	0.0346 (7)	0.0382 (8)	−0.0067 (6)	0.0114 (7)	−0.0024 (6)
C12	0.0482 (9)	0.0353 (8)	0.0401 (9)	−0.0015 (6)	0.0125 (7)	0.0036 (6)
C13	0.0858 (14)	0.0397 (9)	0.0481 (11)	−0.0105 (9)	0.0095 (10)	−0.0066 (8)
C14	0.0328 (7)	0.0342 (7)	0.0472 (9)	0.0001 (6)	0.0033 (6)	0.0046 (6)
C15	0.0430 (9)	0.0484 (9)	0.0504 (10)	0.0018 (7)	0.0084 (7)	0.0019 (8)
C16	0.0641 (13)	0.0635 (12)	0.0624 (13)	0.0120 (10)	0.0268 (10)	0.0099 (10)
C17	0.0427 (11)	0.0805 (15)	0.099 (2)	0.0087 (10)	0.0262 (12)	0.0261 (14)
C18	0.0370 (10)	0.0903 (17)	0.0909 (19)	−0.0082 (10)	0.0017 (10)	0.0159 (14)
C19	0.0393 (9)	0.0626 (11)	0.0643 (13)	−0.0084 (8)	−0.0046 (8)	0.0023 (10)
C20	0.0407 (8)	0.0355 (7)	0.0342 (8)	−0.0041 (6)	0.0039 (6)	−0.0051 (6)
C21	0.0396 (9)	0.0625 (11)	0.0495 (10)	−0.0061 (8)	0.0085 (7)	−0.0077 (8)
C22	0.0401 (9)	0.0656 (12)	0.0581 (12)	0.0034 (8)	−0.0046 (8)	−0.0095 (9)
C23	0.0586 (11)	0.0491 (10)	0.0407 (9)	0.0097 (8)	−0.0084 (8)	−0.0021 (7)
C24	0.0520 (10)	0.0486 (9)	0.0377 (9)	0.0037 (7)	0.0063 (7)	0.0011 (7)
C25	0.0368 (8)	0.0380 (7)	0.0352 (8)	−0.0002 (6)	0.0031 (6)	−0.0031 (6)
N1	0.0413 (7)	0.0368 (6)	0.0322 (7)	−0.0038 (5)	−0.0001 (5)	−0.0018 (5)
N2	0.0401 (8)	0.0659 (10)	0.0436 (8)	−0.0019 (7)	0.0071 (6)	0.0000 (7)
O1	0.0898 (10)	0.0357 (6)	0.0513 (8)	−0.0036 (6)	0.0053 (7)	0.0092 (5)
O2	0.1389 (17)	0.0368 (7)	0.0692 (11)	−0.0122 (8)	0.0074 (10)	−0.0030 (7)
O3	0.0634 (8)	0.0438 (6)	0.0469 (7)	0.0007 (6)	0.0015 (6)	−0.0146 (5)
O4	0.0353 (6)	0.0406 (6)	0.0492 (7)	0.0047 (4)	−0.0024 (5)	−0.0001 (5)
O5	0.0471 (8)	0.1079 (13)	0.0652 (10)	0.0121 (8)	0.0009 (7)	0.0162 (9)
O6	0.0562 (10)	0.1501 (19)	0.1014 (14)	−0.0264 (11)	0.0103 (9)	0.0524 (14)
F1	0.0809 (9)	0.0987 (10)	0.0484 (7)	0.0167 (7)	−0.0194 (6)	0.0070 (7)
S1	0.03481 (19)	0.03234 (18)	0.0381 (2)	0.00070 (14)	0.00198 (14)	−0.00437 (14)

### Geometric parameters (Å, º)

**Table:**

C1—C2	1.384 (2)	C14—S1	1.7459 (18)
C1—C6	1.395 (2)	C15—C16	1.384 (3)
C1—N1	1.427 (2)	C15—H15	0.9300
C2—C3	1.383 (3)	C16—C17	1.385 (4)
C2—H2	0.9300	C16—H16	0.9300
C3—C4	1.370 (3)	C17—C18	1.359 (4)
C3—H3	0.9300	C17—H17	0.9300
C4—C5	1.375 (3)	C18—C19	1.374 (3)
C4—H4	0.9300	C18—H18	0.9300
C5—C6	1.395 (2)	C19—H19	0.9300
C5—H5	0.9300	C20—C25	1.387 (2)
C6—C7	1.437 (2)	C20—C21	1.394 (2)
C7—C12	1.390 (2)	C21—C22	1.373 (3)
C7—C8	1.396 (2)	C21—H21	0.9300
C8—C9	1.390 (2)	C22—C23	1.368 (3)
C8—N1	1.4117 (19)	C22—H22	0.9300
C9—C10	1.370 (2)	C23—F1	1.345 (2)
C9—H9	0.9300	C23—C24	1.370 (3)
C10—C11	1.414 (2)	C24—C25	1.375 (2)
C10—C20	1.490 (2)	C24—H24	0.9300
C11—C12	1.402 (2)	C25—N2	1.467 (2)
C11—C13	1.437 (2)	N1—S1	1.6608 (14)
C12—O1	1.3411 (19)	N2—O5	1.202 (2)
C13—O2	1.218 (2)	N2—O6	1.209 (2)
C13—H13	0.9300	O1—H1	0.8200
C14—C15	1.381 (3)	O3—S1	1.4189 (12)
C14—C19	1.384 (2)	O4—S1	1.4202 (13)
			
C2—C1—C6	121.76 (15)	C16—C15—H15	120.7
C2—C1—N1	130.13 (16)	C15—C16—C17	119.5 (2)
C6—C1—N1	108.11 (13)	C15—C16—H16	120.2
C3—C2—C1	116.81 (18)	C17—C16—H16	120.2
C3—C2—H2	121.6	C18—C17—C16	121.0 (2)
C1—C2—H2	121.6	C18—C17—H17	119.5
C4—C3—C2	122.32 (18)	C16—C17—H17	119.5
C4—C3—H3	118.8	C17—C18—C19	120.5 (2)
C2—C3—H3	118.8	C17—C18—H18	119.8
C3—C4—C5	120.94 (17)	C19—C18—H18	119.8
C3—C4—H4	119.5	C18—C19—C14	118.7 (2)
C5—C4—H4	119.5	C18—C19—H19	120.6
C4—C5—C6	118.34 (18)	C14—C19—H19	120.6
C4—C5—H5	120.8	C25—C20—C21	116.44 (16)
C6—C5—H5	120.8	C25—C20—C10	122.55 (15)
C1—C6—C5	119.77 (16)	C21—C20—C10	120.77 (15)
C1—C6—C7	107.50 (14)	C22—C21—C20	121.61 (17)
C5—C6—C7	132.61 (16)	C22—C21—H21	119.2
C12—C7—C8	118.84 (15)	C20—C21—H21	119.2
C12—C7—C6	132.79 (15)	C23—C22—C21	118.93 (17)
C8—C7—C6	108.34 (14)	C23—C22—H22	120.5
C9—C8—C7	122.70 (14)	C21—C22—H22	120.5
C9—C8—N1	129.30 (14)	F1—C23—C22	119.16 (18)
C7—C8—N1	107.94 (13)	F1—C23—C24	118.45 (18)
C10—C9—C8	117.92 (14)	C22—C23—C24	122.38 (18)
C10—C9—H9	121.0	C23—C24—C25	117.19 (17)
C8—C9—H9	121.0	C23—C24—H24	121.4
C9—C10—C11	121.16 (15)	C25—C24—H24	121.4
C9—C10—C20	117.47 (14)	C24—C25—C20	123.41 (16)
C11—C10—C20	121.34 (14)	C24—C25—N2	115.91 (15)
C12—C11—C10	119.74 (14)	C20—C25—N2	120.66 (15)
C12—C11—C13	119.79 (16)	C8—N1—C1	107.92 (12)
C10—C11—C13	120.40 (16)	C8—N1—S1	124.30 (11)
O1—C12—C7	118.61 (15)	C1—N1—S1	124.56 (11)
O1—C12—C11	121.96 (15)	O5—N2—O6	122.76 (17)
C7—C12—C11	119.40 (14)	O5—N2—C25	119.13 (15)
O2—C13—C11	125.2 (2)	O6—N2—C25	118.10 (16)
O2—C13—H13	117.4	C12—O1—H1	109.5
C11—C13—H13	117.4	O3—S1—O4	119.96 (7)
C15—C14—C19	121.56 (17)	O3—S1—N1	106.50 (7)
C15—C14—S1	119.38 (13)	O4—S1—N1	106.35 (7)
C19—C14—S1	119.04 (15)	O3—S1—C14	109.70 (8)
C14—C15—C16	118.67 (18)	O4—S1—C14	108.93 (8)
C14—C15—H15	120.7	N1—S1—C14	104.19 (7)
			
C6—C1—C2—C3	−2.3 (3)	C15—C14—C19—C18	0.5 (3)
N1—C1—C2—C3	177.26 (16)	S1—C14—C19—C18	−177.66 (16)
C1—C2—C3—C4	1.4 (3)	C9—C10—C20—C25	−75.8 (2)
C2—C3—C4—C5	0.8 (3)	C11—C10—C20—C25	102.15 (19)
C3—C4—C5—C6	−1.9 (3)	C9—C10—C20—C21	98.30 (19)
C2—C1—C6—C5	1.2 (2)	C11—C10—C20—C21	−83.8 (2)
N1—C1—C6—C5	−178.45 (14)	C25—C20—C21—C22	−0.4 (3)
C2—C1—C6—C7	177.77 (15)	C10—C20—C21—C22	−174.86 (17)
N1—C1—C6—C7	−1.89 (17)	C20—C21—C22—C23	1.0 (3)
C4—C5—C6—C1	0.9 (2)	C21—C22—C23—F1	179.39 (18)
C4—C5—C6—C7	−174.60 (17)	C21—C22—C23—C24	0.0 (3)
C1—C6—C7—C12	−178.73 (17)	F1—C23—C24—C25	179.14 (16)
C5—C6—C7—C12	−2.8 (3)	C22—C23—C24—C25	−1.4 (3)
C1—C6—C7—C8	−0.87 (17)	C23—C24—C25—C20	2.0 (3)
C5—C6—C7—C8	175.08 (16)	C23—C24—C25—N2	−179.38 (16)
C12—C7—C8—C9	4.2 (2)	C21—C20—C25—C24	−1.1 (2)
C6—C7—C8—C9	−174.01 (14)	C10—C20—C25—C24	173.19 (16)
C12—C7—C8—N1	−178.49 (13)	C21—C20—C25—N2	−179.64 (15)
C6—C7—C8—N1	3.30 (16)	C10—C20—C25—N2	−5.3 (2)
C7—C8—C9—C10	0.0 (2)	C9—C8—N1—C1	172.62 (15)
N1—C8—C9—C10	−176.72 (14)	C7—C8—N1—C1	−4.45 (16)
C8—C9—C10—C11	−4.0 (2)	C9—C8—N1—S1	12.2 (2)
C8—C9—C10—C20	173.94 (14)	C7—C8—N1—S1	−164.88 (11)
C9—C10—C11—C12	3.8 (2)	C2—C1—N1—C8	−175.68 (16)
C20—C10—C11—C12	−174.03 (15)	C6—C1—N1—C8	3.93 (16)
C9—C10—C11—C13	−179.24 (16)	C2—C1—N1—S1	−15.3 (2)
C20—C10—C11—C13	2.9 (2)	C6—C1—N1—S1	164.29 (11)
C8—C7—C12—O1	177.74 (14)	C24—C25—N2—O5	147.23 (18)
C6—C7—C12—O1	−4.6 (3)	C20—C25—N2—O5	−34.1 (2)
C8—C7—C12—C11	−4.3 (2)	C24—C25—N2—O6	−33.4 (3)
C6—C7—C12—C11	173.39 (16)	C20—C25—N2—O6	145.2 (2)
C10—C11—C12—O1	178.36 (15)	C8—N1—S1—O3	−165.23 (12)
C13—C11—C12—O1	1.4 (3)	C1—N1—S1—O3	37.55 (14)
C10—C11—C12—C7	0.5 (2)	C8—N1—S1—O4	−36.20 (14)
C13—C11—C12—C7	−176.50 (16)	C1—N1—S1—O4	166.58 (12)
C12—C11—C13—O2	−0.9 (3)	C8—N1—S1—C14	78.81 (14)
C10—C11—C13—O2	−177.9 (2)	C1—N1—S1—C14	−78.41 (14)
C19—C14—C15—C16	−1.4 (3)	C15—C14—S1—O3	166.60 (13)
S1—C14—C15—C16	176.77 (14)	C19—C14—S1—O3	−15.16 (16)
C14—C15—C16—C17	1.1 (3)	C15—C14—S1—O4	33.48 (15)
C15—C16—C17—C18	0.1 (3)	C19—C14—S1—O4	−148.28 (14)
C16—C17—C18—C19	−1.0 (4)	C15—C14—S1—N1	−79.71 (14)
C17—C18—C19—C14	0.7 (3)	C19—C14—S1—N1	98.53 (14)

### Hydrogen-bond geometry (Å, º)

**Table:**

*D*—H···*A*	*D*—H	H···*A*	*D*···*A*	*D*—H···*A*
O1—H1···O2	0.82	1.89	2.611 (3)	146
C2—H2···O3	0.93	2.37	2.956 (3)	121
C9—H9···O4	0.93	2.30	2.902 (3)	122
C18—H18···O4^i^	0.93	2.55	3.221 (3)	129
C13—H13···O4^ii^	0.93	2.50	3.337 (3)	150

Symmetry codes: (i) *x*−1, *y*, *z*; (ii) −*x*+1, *y*+1/2, −*z*+1/2.
